# Dynamics of nitric oxide level in liquids treated with microwave plasma-generated gas and their effects on spinach development

**DOI:** 10.1038/s41598-018-37711-3

**Published:** 2019-01-30

**Authors:** Min Ho Kang, Seong Sil Jeon, So Min Shin, Mayura Veerana, Sang-Hye Ji, Han-Sup Uhm, Eun-Ha Choi, Jae Ho Shin, Gyungsoon Park

**Affiliations:** 1 Plasma Bioscience Research Center, Kwangwoon University, Seoul, 01897 Korea; 2 Department of Electrical and Biological Physics, Kwangwoon University, Seoul, 01897 Korea; 3 Department of Chemistry, Kwangwoon University, Seoul, 01897 Korea; 4 New Industry Convergence Technology R&D Center, Ajou University, Suwon, 16499 Korea; 5 Present Address: Plasma Technology Research Center, National Fusion Research Institute, Gunsan-si, Jeollabuk-Do, 54004 Republic of Korea

## Abstract

In this study, we generated water and phosphate buffer treated with microwave plasma-generated gas in which the major component was nitric oxide (PGNO), and investigated the efficiency of the treated water and buffer in fertilization and sanitation. Real time NO level monitored by an electrode sensor was linearly increased over PGNO injection time, and removal of O_2_ from liquid before PGNO injection accelerated NO assimilation into liquids. Residual NO was still present 16 h after PGNO injection was stopped. H_2_O_2_, NO_2_^−^, and NO_3_^−^ were also detected in PGNO-treated liquids. Spinach plants applied with 10 and 30 times diluted PGNO-treated water and 0.5 mM phosphate buffer showed slightly higher height and dry weight than control after 5 weeks. Plants grown with 10 and 30 times diluted PGNO-treated water exhibited the increased tolerance to water deficiency. Significant anti-microbial activity within 1 h was observed in un-diluted and in half-diluted PGNO-treated water and 0.5 mM phosphate buffer. Our results suggest that water or phosphate buffer containing NO, H_2_O_2_, NO_2_^−^, and NO_3_^−^ can be produced by PGNO treatment, and that PGNO-treated water or buffer can be used as a potential fertilizer enhancing plant vitality with sanitation effect.

## Introduction

Indoor and greenhouse cultivation have been frequently practiced, in order to reduce the influence of climatic variation^1,2^. In these practices, water, fertilization and sanitation are important factors in determining the success of crop cultivation. Chemical based inorganic and organic fertilization have been routinely used to enhance crop growth and yield^3–5^. However, problems have recently been emerging, such as the pollution of underground water and environment with residual phosphorus (P) and nitrogen (N) left in soil^6^. Alternative strategies should be considered to establish safe and efficient cultivation practices under unpredictable environmental conditions.

Plasma-treated water or media have been shown to be effective in fertilization and sanitation^7–11^. Plasma is an ionized gas that is often known as the 4^th^ state of matter^12^. In particular, non-thermal plasma produced at atmospheric pressure is known to have effects of microbial inactivation and tissue regeneration, and reactive oxygen and nitrogen species generated by plasma play an important role in exerting these biological effects^13–15^. Although the action mechanisms of plasma-treated water or media still need intensive research, reactive species dissolved in plasma-treated water or media may be major players in the sterilization and regeneration processes^16–19^.

Nitric oxide (NO), one of the reactive nitrogen species generated by plasma, is well known to regulate resistance to abiotic and biotic stresses, and development, in plant^20,21^. It is a potentially highly reactive species that can generate contrary effects on plant (activation) and microbes (inactivation). Plants produce intracellular NO, and intracellular NO is involved in activating defense signal transduction in response to pathogen infection and drought stress in plants^22–24^. Recently, it has been found to control plant germination and growth^21,25^. Exogenous NO is applied to plants in order to enhance plant health and stress regulation, as well as microbial inactivation^21^. Pure NO gas and NO producing chemicals and nanoparticles have been used as sources for exogenous NO, but limitations in using these sources have also been found in terms of efficiency and economic costs^26–28^. Plasma (particularly microwave plasma) generated NO can be another potential source for exogenous NO, which can be produced in large quantity with less cost compared to pure NO gas. A study shows that NO dominant in a plasma jet generated using air and DC voltage is responsible for the inactivation of yeast and bacteria^29^. Plasma-generated NO as a potential fertilizer and enhancer of plant vitality has not been actively studied. In particular, fertilization that makes plants stronger to stresses caused by climate change is becoming more important these days than ever. In this study, we investigated the potentiality of water and buffer treated with microwave plasma-generated gas, in which the major component was NO, in enhancing plant vitality and inactivating microorganisms. Particularly, relationship with the chemistry of water and buffer treated with microwave plasma-generated gas was intensely examined in the study.

## Results

### Level of NO dissolved in water and phosphate buffer

**Figure 1:**
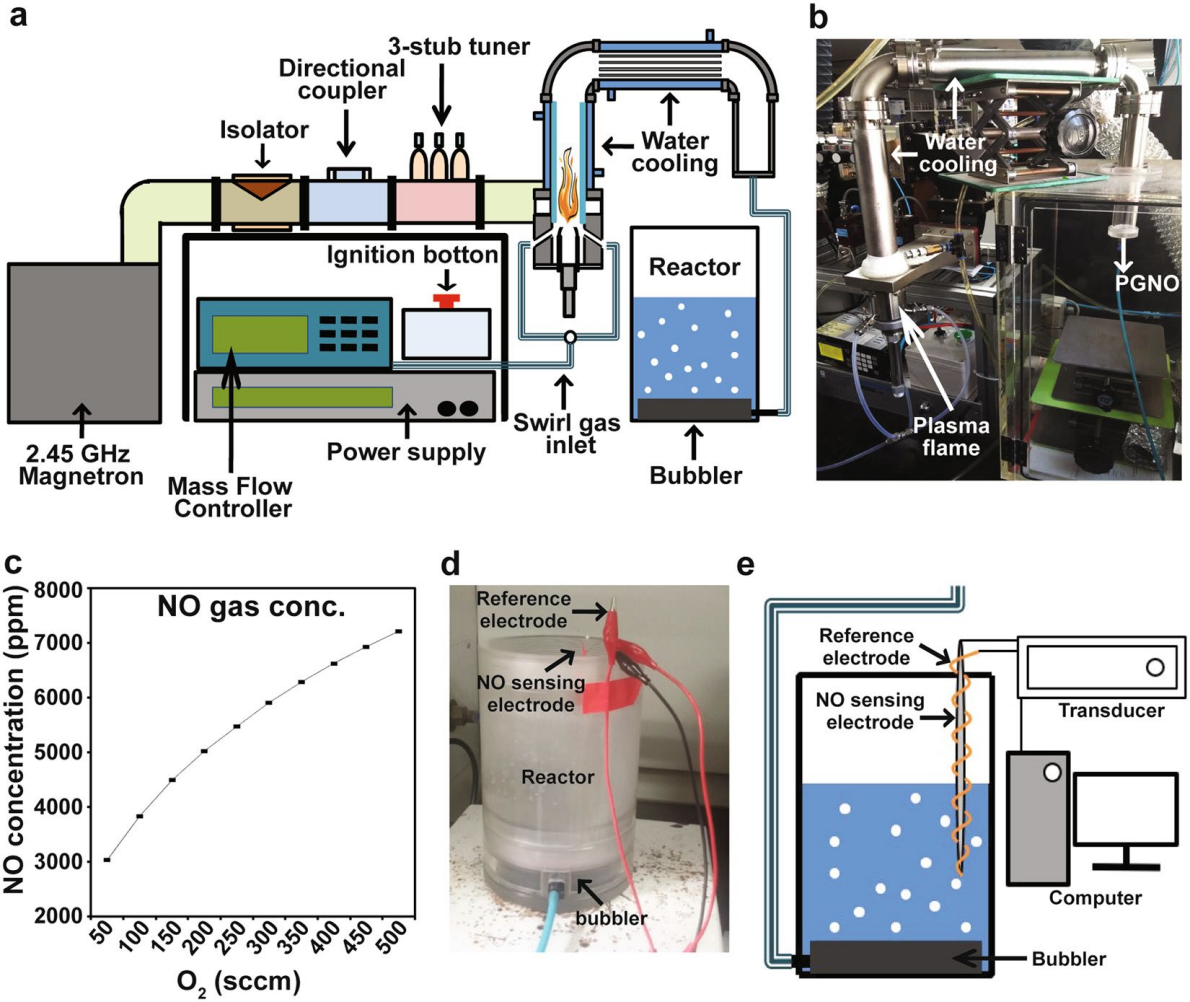
Treatment with plasma-generated nitric oxide (PGNO) and measurement of real time NO concentration. (**a**) Schematic of the microwave plasma torch system and PGNO injection into water (1 L) and phosphate buffer (1 L). (**b**) Picture of the microwave plasma torch system. (**c**) Concentration of gaseous NO measured using NO gas measurement instrument. (**d**) Set-up of electrodes for sensing real time NO in liquid. (**e**) Schematic view of electrodes sensor system.

In our study, microwave torch plasma was produced using the mixture of nitrogen and oxygen gas provided with 10 lpm (liter per minute) and 400 sccm (cc per minute), respectively (Fig. 1a and b). The level of nitric oxide (NO) in gas generated by microwave plasma torch was increased when more oxygen was provided per unit time (Fig. 1c). About 6,500 ppm of nitric oxide (NO) was detected in the plasma-generated gas produced using 10 lpm nitrogen and 400 sccm oxygen (Fig. 1c). Since the majority of gas generated by microwave plasma torch was nitric oxide (NO), we designated the gas as plasma-generated nitric oxide (PGNO) in further description in the text.

**Figure 2:**
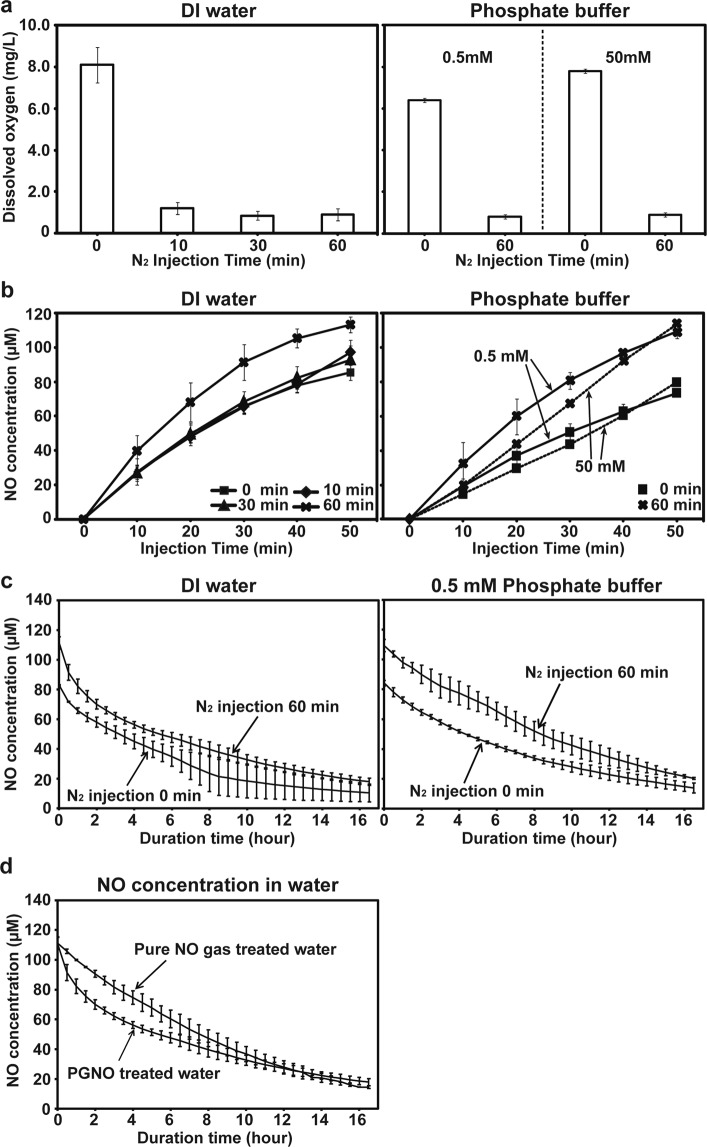
Generation and decay of NO in DI water and phosphate buffer treated with PGNO. (**a**) Concentration of dissolved oxygen in DI water and potassium phosphate buffer (0.5 and 50 mM, pH 6.0) after N_2_ gas injection. Concentration of O_2_ dissolved in DI (deionized) water and potassium phosphate buffer (0.5 and 50 mM) was measured, after injecting N_2_ gas for indicated time. (**b**) Level of NO dissolved in PGNO-treated water and phosphate buffer over different PGNO injection time. PGNO was injected after O_2_ was purged by N_2_ injection for 0, 10, 30, or 60 min. (**c**) Change in level of NO dissolved in PGNO-treated water and 0.5 mM phosphate buffer over time, after PGNO injection was stopped. (**d**) Decay of NO in DI water treated with pure NO gas and PGNO. PGNO was injected into 1 L of DI water for 50 min without O_2_ purging, and pure NO gas was injected until NO level was similar to that in PGNO-treated water.

After PGNO was injected into 1 L of deionized water or 0.5 and 50 mM potassium phosphate buffer (pH 6.0), the level of dissolved nitric oxide was measured in real time using electrode sensor (Fig. 1d and e). Since oxygen dissolved in water and phosphate buffer can react with nitric oxide, resulting in the reduction of nitric oxide level in liquids, it was purged by injecting nitrogen gas into water and buffer, before injecting PGNO. The concentration of oxygen dissolved in water was dramatically reduced after the injection of nitrogen gas (Fig. 2a). About 90% of the dissolved oxygen was purged after 10 min of nitrogen gas injection, and the level of dissolved oxygen was not significantly changed after that (Fig. 2a). Likewise, about 90% of dissolved oxygen in both 0.5 and 50 mM potassium phosphste buffer was removed after injection of nitrogen gas for 60 min (Fig. 2a). The NO microsensor modified with perfluorinated xerogel-derived gas-permeable membrane (i.e., a membrane selectively permeable towards NO) was characterized by 27.5 nA/μM of sensitivity, 0.1 μM of detection limit (S/N = 3), and < 3 s of response time (*t*_95%_) with negligible response towards interfering species (at 50 μM for each species) such as hydrogen peroxide (H_2_O_2_), nitrite (NO_2_^−^), nitrate (NO_3_^−^), peroxynitrite (ONOO^−^), hydroxyl radical (OH·), and superoxide (O_2_^−^) (Supplementary Fig. S1). The level of nitric oxide (NO) dissolved in water and phosphate buffer was increased over the injection time of PGNO (Fig. 2b). In water, nitric oxide level was more rapidly increased when oxygen was purged for 60 min (Fig. 2b). In potassium phosphate buffer, the NO level was increased faster with oxygen purging, than without (Fig. 2b). The NO concentration was increased slightly faster in 0.5 mM than in 50 mM phosphate buffer, but the level became similar after PGNO injection for 50 min (Fig. 2b). Generally, the increase in NO level was slightly faster in water than phosphate buffer under similar level of oxygen purging (Fig. 2b).

The level of NO was continuously monitored after the PGNO injection was ceased. After PGNO was injected for 50 min and then ceased, the NO concentration was exponentially decreased over time, and about 15–30 µM NO was detected in water and phosphate buffer after 16 h (Fig. 2c). There was no significant difference in the rate of NO level decrease between with and without oxygen purging (Fig. 2c). The dynamics of NO decay in PGNO injected water was compared to that in pure NO gas (commercially purchased) injected water, in order to determine NO life in 1 L water. Pure NO gas was injected into 1 L water (no oxygen purging), until the dissolved NO level reached that of PGNO injected (for 50 min) water. After the pure NO gas and PGNO injection was stopped, the NO level was decreased slightly faster in PGNO than pure NO gas injected water in the early stage, and then decreased at similar rate in both liquids after 10 h (Fig. 2d). The NO level reached around 20 µM in both pure NO gas and PGNO injected water after 16 h (Fig. 2d).

**Figure 3:**
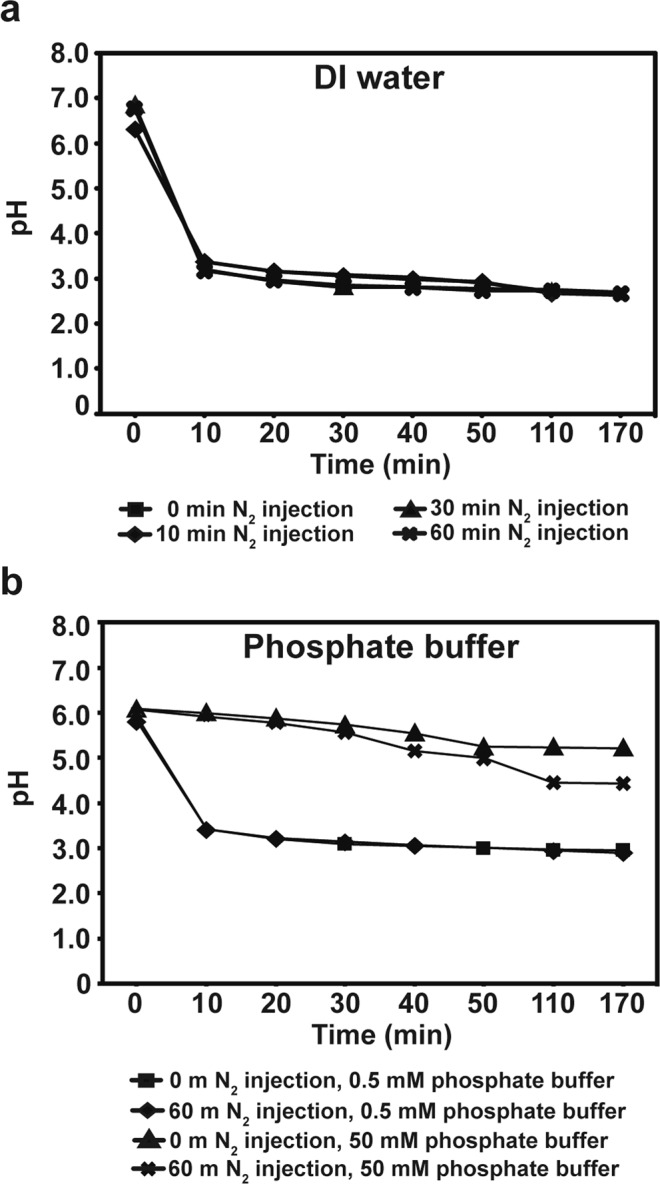
The pH of PGNO-treated DI water and phosphate buffer. (**a**) The pH of DI water monitored during and after PGNO injection. (**b**) The pH of potassium phosphate buffer (0.5 mM and 50 mM) monitored during and after PGNO injection. In (**a**) and (**b**), PGNO was injected for 50 min. Before PGNO injection, N_2_ was injected for 0, 10, 30, or 60 min to remove O_2_. All data points represent mean ± standard deviation of 3 replicate measurements.

The pH of both water and 0.5 mM phosphate buffer was dramatically reduced to around 3 after PGNO injection for 10 min, and then no further change was observed during PGNO injection. About pH 3 was maintained up to 2 h after stopping PGNO injection (Fig. 3). In 50 mM phosphate buffer, the pH was slightly reduced during PGNO injection (Fig. 3). The pH was around 5.5 after PGNO injection for 50 min, and similar pH was kept up to 2 h without PGNO injection (Fig. 3). This might be due to the higher buffering strength of 50 mM phosphate buffer than water and 0.5 mM buffer. Oxygen purging did not change the pattern of pH decrease in both water and phosphate buffers (Fig. 3).

### Level of other reactive species and ions in water and phosphate buffer

**Figure 4:**
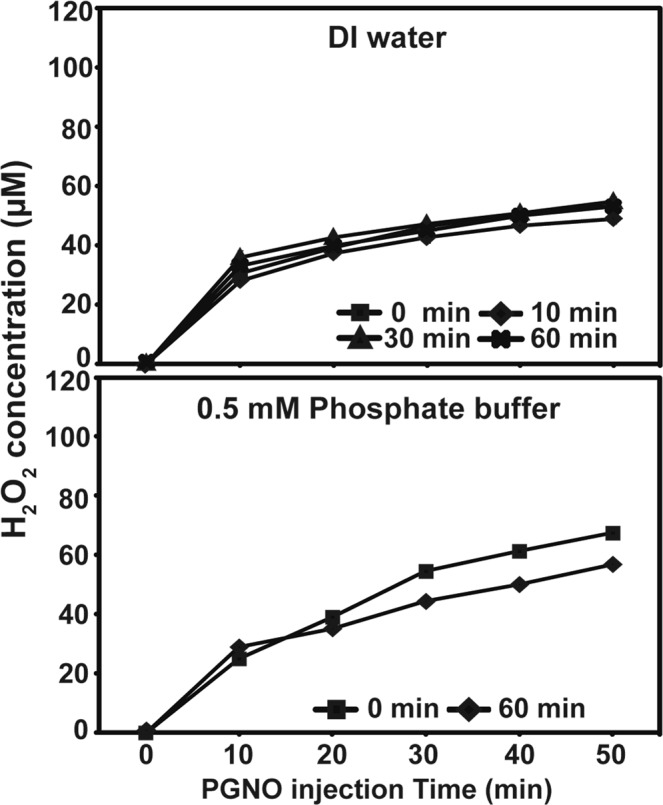
Generation of H_2_O_2_ in DI water and 0.5 mM phosphate buffer during PGNO injection. Change in concentration of H_2_O_2_ in DI water and 0.5 mM potassium phosphate buffer (pH 6.0) during PGNO injection. PGNO was injected after O_2_ purging by injecting N_2_ gas for 0, 10, 30, and 60 min.

We observed that the NO level was decreased slightly faster in PGNO than pure NO gas injected water in the early stage (Fig. 2d). This suggests that NO has reacted with other species that may be present in PGNO water. To test this hypothesis, we investigated the presence of other reactive and ion species in PGNO treated water and buffer. Hydrogen peroxide (H_2_O_2_) was detected in PGNO-treated water and phosphate buffer. The H_2_O_2_ microsensor electropolymerized with poly(3-aminobenzoic acid) (PABA) permselective membrane (i.e., a membrane selectively permeable towards H_2_O_2_) was characterized by 3.15 nA/μM of sensitivity, 0.6 μM of detection limit (S/N = 3), and *t*_95%_ < 3 s of response time with negligible response towards interfering species (at 50 μM for each species) such as nitric oxide (NO), nitrite (NO_2_^−^), nitrate (NO_3_^−^), peroxynitrite (ONOO^−^), hydroxyl radical (OH), and superoxide (O_2_^−^) (Supplementary Fig. S2). H_2_O_2_ level was gradually increased during PGNO injection, but not as rapidly as that of NO (Fig. 4). About 50–65 µM H_2_O_2_ was detected in both water and 0.5 mM phosphate buffer after 50 min PGNO injection (Fig. 4). Oxygen purging did not change the H_2_O_2_ level in water (Fig. 4). However, in 0.5 mM phosphate buffer, the H_2_O_2_ level increased slightly faster without oxygen purging, than with (Fig. 4). The presence of several ions was analyzed in PGNO-treated water and phosphate buffer using ion chromatography. None of the positive ions analyzed was detected in both PGNO-treated water and phosphate buffer, except potassium (from potassium phosphate buffer) (Table 1). For negative ions, nitrite (NO_2_^−^) and nitrate (NO_3_^−^) were detected in PGNO-treated water and phosphate buffer, besides phosphate ion (PO_4_^3−^) from potassium phosphate (Table 1). About 30–40 ppm NO_2_^−^ and 110–140 ppm NO_3_^−^ were measured in both water and phosphate buffer treated with PGNO for 50 min (Table 1). No significant difference in NO_2_^−^ and NO_3_^−^ level was observed between different oxygen purging time (Table 1).

**Table 1:** Concentration of ions in PGNO treated DI water and 0.5 mM phosphate buffer.

Ions	DI water (mg/L)					0.5 mM phosphate buffer (mg/L)		
	Non-treated	PGNO-treated				Non-treated	PGNO-treated	
		N_2_ injection (min)					N_2_ injection (min)	
		0	10	30	60		0	60
**Anion**								
Cl	0.0109*	0.0612	0.0792	n.d.**	0.0414	0.0041	0.0589	0.0555
NO_2_	n.d.	39.66	33.34	35.08	38.97	0.0014	33.19	30.66
NO_3_	n.d.	130.38	128.87	110.20	137.58	n.d.	113.23	130.21
SO_4_	0.2936	0.235	0.242	0.254	0.294	0.138	0.243	0.242
PO_4_	0.0673	0.369	0.091	n.d.	n.d.	31.61	24.06	31.48
**Cation**								
Li	n.d.	0.105	0.105	0.105	0.105	0.105	0.105	0.105
Na	n.d.	n.d.	0.249	0.486	0.586	n.d.	0.931	0.843
NH_4_	n.d.	n.d.	n.d.	n.d.	n.d.	n.d.	n.d.	n.d.
K	n.d.	0.821	0.692	0.693	0.683	12.83	10.30	13.61
Mg	n.d.	0.0532	0.0564	0.0506	0.0536	0.0471	0.0561	0.0565
Ca	0.773	0.830	0.851	0.813	0.841	0.786	0.844	0.852

*Concentrtion of ion in DI water and 0.5 mM potassium phosphate buffer (pH 6.0) treated with PGNO for 50 min. Before PGNO treatment, O_2_ was removed by N_2_ injection for 0, 10, 30, or 60 min.

**Not detectable.

### Effect of PGNO-treated water and phosphate buffer on plant vitality

**Figure 5:**
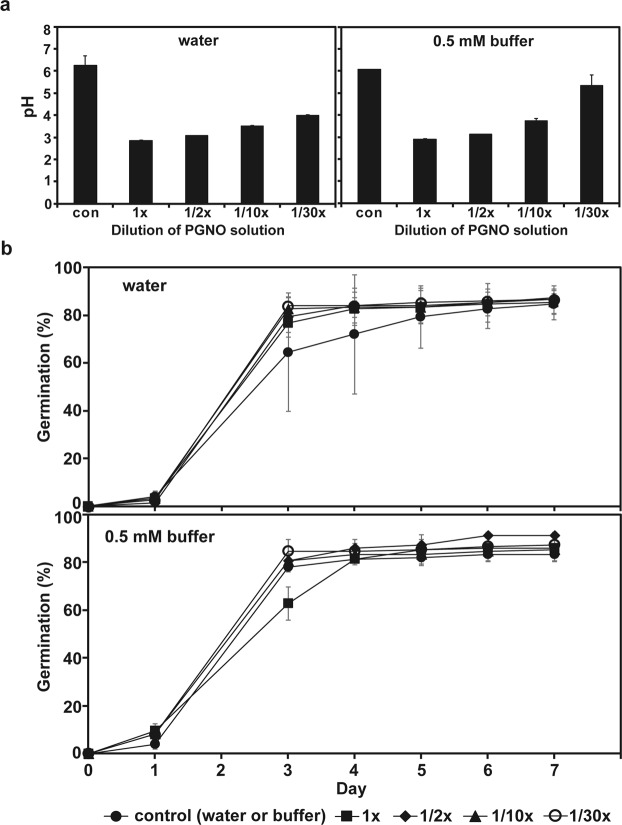
The pH of PGNO-treated DI water and 0.5 mM phosphate buffer with different dilution and germination of spinach seeds. (**a**) The pH of differently diluted DI water and 0.5 mM potassium phosphate buffer (pH 6.0) treated with or without (control) PGNO. Dilution was done 0 (1×), 2 (1/2×), 10 (10×), and 30 (30x) times. (**b**) Germination of spinach seeds assessed for 7 days after seeds were soaked in PGNO-treated DI water and 0.5 mM potassium phosphate buffer (pH 6.0) with different dilutions for 30 min. Control indicates no PGNO-treated water and buffer. All measurements were performed in 3 replicates.

PGNO-treated (50 min) water and 0.5 mM phosphate buffer without and with dilution of 2, 10, and 30 times were applied to spinach seeds, and the germination and development were analyzed. The pH of PGNO-treated water and phosphate buffer was generally lower than that of control (no PGNO treatment), ranging from pH 3 to pH 5.5 (Fig. 5a, Supplementary Table S1). The number of spinach seeds germinated after being soaked in PGNO-treated water or phosphate buffer for 30 min increased over time, and about 85% germination was acquired after 3 days (Fig. 5b, Supplementary Table S1). No significant difference in the speed and percentage of seed germination was observed between control and PGNO-treated solutions, and about 85–90% of seeds were germinated after 7 days in all treatments (Fig. 5b).

**Figure 6:**
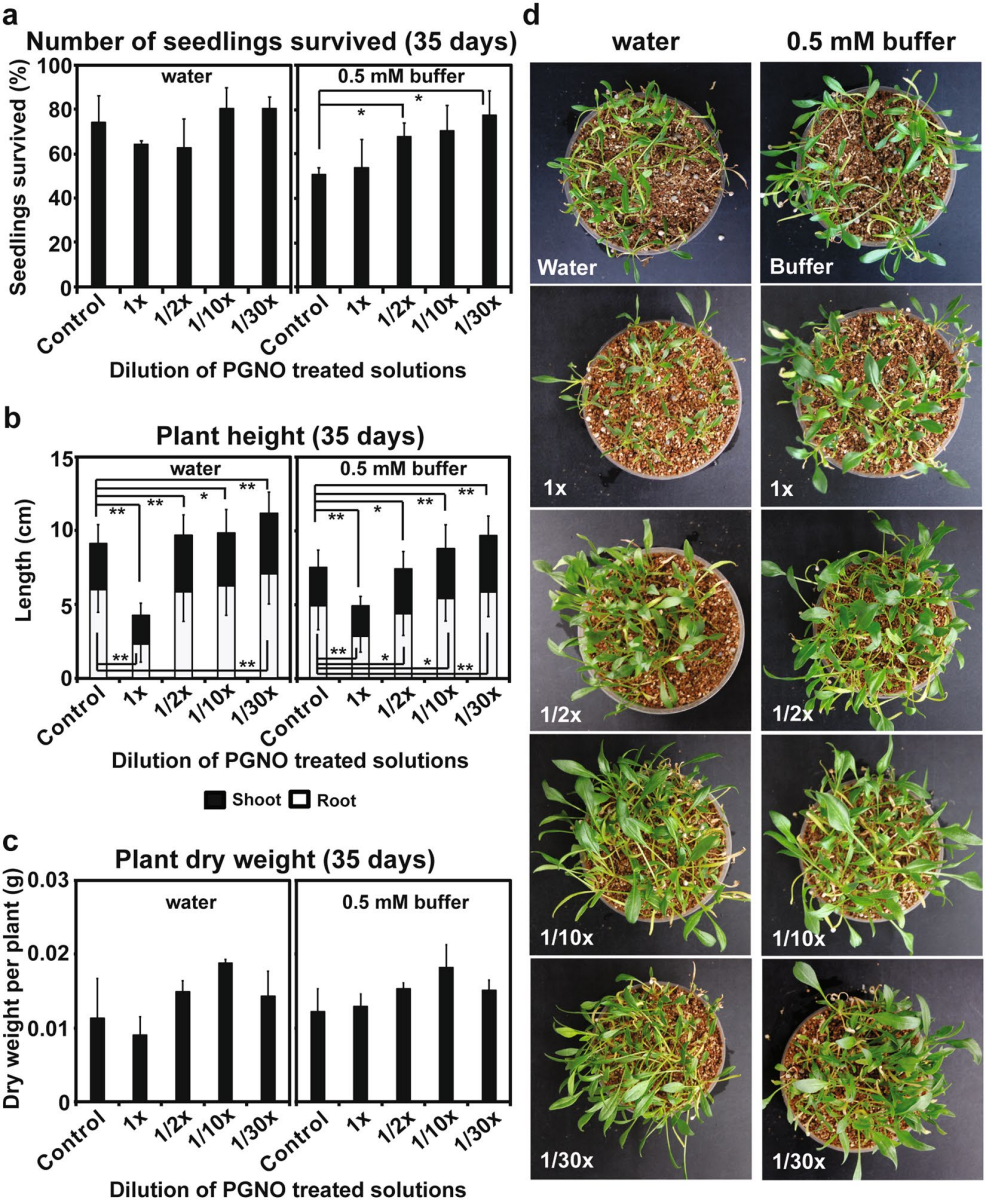
Effect of PGNO-treated DI water and 0.5 mM phosphate buffer on spinach growth. (**a**) Number of seedlings survived after 35 days post-treatment with differently diluted PGNO water and phosphate buffer. Fifty seeds were used in each treatment and percentage of survived seedlings number after 35 days was calculated. The measurement was repeated 3 times and averaged. These 3 repeated measurements were used for statistical analysis. *p < 0.05. (**b**) Average shoot and root length of individual plant grown for 35 days after treatment with differently diluted PGNO water and phosphate buffer. Length was individually measured from 76–121 plants per treatment, and all measurements were used for statistical analysis. *p < 0.05, **p < 0.01. (**c**) Average dry weight of individual plant grown for 35 days after treatment with differently diluted PGNO water and phosphate buffer. Total dry weight of 22–45 plants per treatment was measured, and the average dry weight of individual plant was calculated by dividing total dry weight by the number of plants. These measurements and calculations were repeated 3 times and averaged again. Three replicates were used for statistical analysis. (**d**) Picture of plants grown for 35 days after treatment with differently diluted PGNO water and phosphate buffer.

Seedling growth was monitored after 35 days (5 weeks). The percentage of survived seedling number after 35 days was significantly greater in the treatment with 1/2 and 30 times diluted phosphate buffer (0.5 mM), than in control (no PGNO-treated buffer) (Fig. 6a, Supplementary Table S1). A slightly higher percentage was observed in the treatment with 10 and 30 times diluted PGNO water and 10 times diluted PGNO buffer (Fig. 6a). The shoot length of plants grown for 35 days was significantly increased after the treatment with 10 and 30 times diluted PGNO water and phosphate buffer (Fig. 6b, Supplementary Table S1). Root length was significantly greater in plants treated with 30 times diluted PGNO water and 10 and 30 times diluted phosphate buffer (Fig. 6b, Supplementary Table S1). Average dry weight per plant was slightly higher in the treatment with 1/2, 10, and 30 times diluted PGNO water and phosphate buffer than control (Fig. 6c, Supplementary Table S1). The highest average dry weight per plant was observed in the treatments with 10 times diluted PGNO water and phosphate buffer (Fig. 6c). Plants treated with no diluted PGNO water and phosphate buffer showed lower survival, height, and dry weight than control (Fig. 6a–d). Figure 6D shows that plants treated with no diluted PGNO-treated water and phosphate buffer have narrower leaves and are poorly grown, compared to control. Plants treated with 10 and 30 times diluted PGNO water and phosphate buffer seem to have slightly larger and more abundant leaves than control (Fig. 6d).

**Figure 7:**
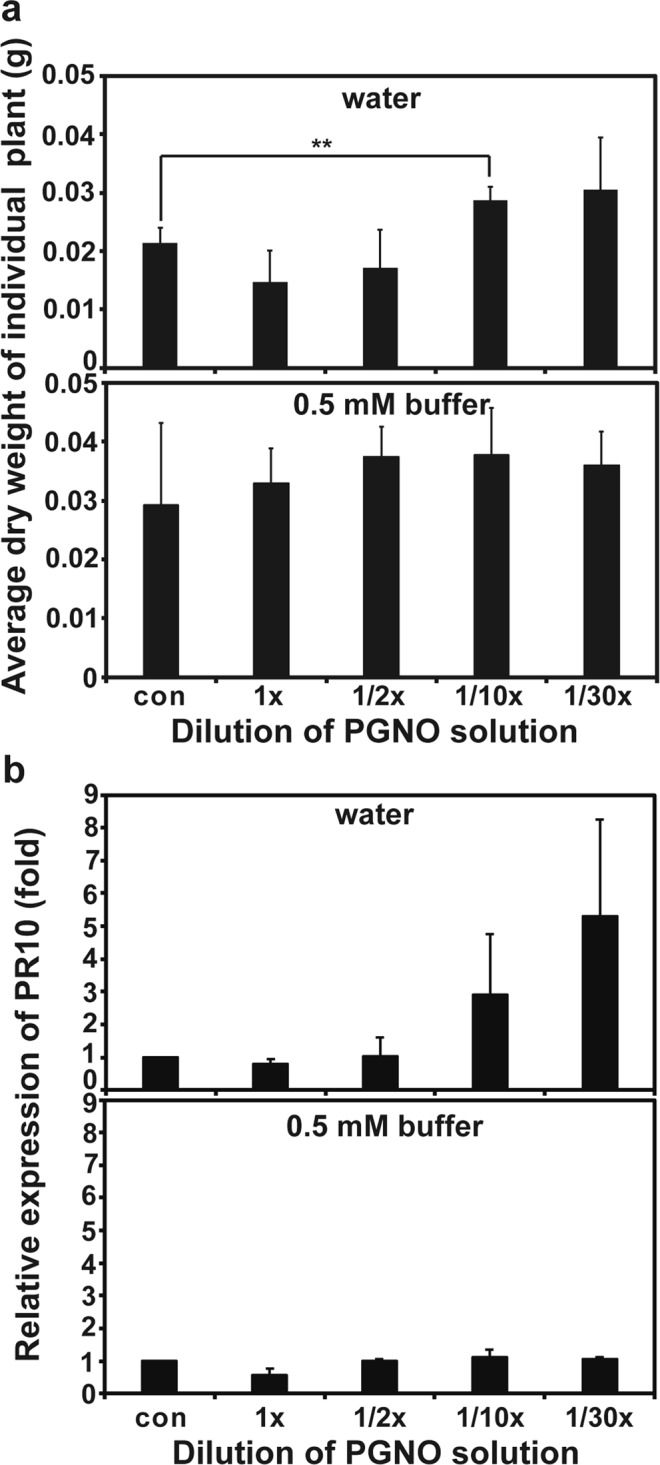
Effect of PGNO-treated DI water and 0.5 mM phosphate buffer on plant stress response. (**a**) Average dry weight of individual plant grown under the treatment with differently diluted PGNO water and phosphate buffer for 4 weeks and then under no water condition for additional 2 weeks. Total dry weight of 21–48 plants per treatment was measured, and the average dry weight of individual plant was calculated by dividing total dry weight by the number of plants. These were repeated 4 times and averaged again. Four replicate measurements were used for statistical analysis. **p < 0.01. (**b**) Relative level of PR10 transcripts in 4 week-old spinach plants treated with differently diluted PGNO water and phosphate buffer, compared to control (plants grown with non-treated DI water and 0.5 mM phosphate buffer). Each value represents the average level of transcript ± standard deviation from 3 replicate independent experiments. Three replicate measurements were used for statistical analysis.

Plants grown under the treatment with PGNO water and buffer were tested for tolerance to drought stress and induction for PR10 expression. PR10 is a stress related gene, and its expression is induced in response to drought, salt, nitrosative, and oxidative stresses in spinach, providing the plants stress tolerance^30^. The average dry weight per plant treated with 10 and 30 times diluted PGNO water was significantly greater than that of control after 10 days under drought stress (no watering) (Fig. 7a, Supplementary Table S1). Plants treated with 1/2, 10, and 30 times diluted PGNO phosphate buffer exhibited slightly higher dry weight than control after growth under no water condition for 10 days (Fig. 7a). The transcription level of PR10 was increased in plants treated with 10 and 30 times diluted PGNO water compared to control although the difference was not statistically significant (Fig. 7b, Supplementary Table S1). There was no obvious difference in PR10 transcription between control and PGNO-treated phosphate buffer (Fig. 7a).

### Anti-microbial activity of PGNO-treated water and phosphate buffer

**Figure 8:**
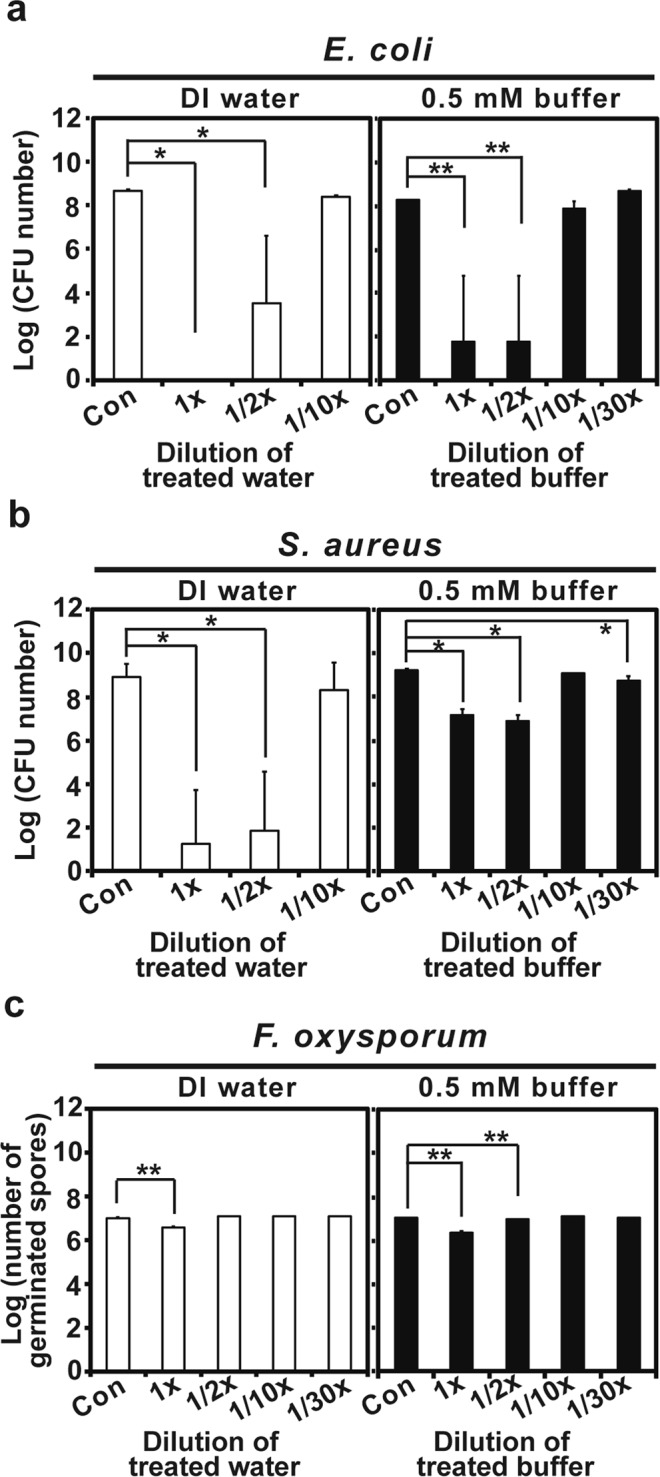
Anti-microbial activity of PGNO-treated DI water and 0.5 mM phosphate buffer. (**a**) CFU number of *E. coli* treated with differently diluted PGNO DI water and 0.5 mM phosphate buffer. Bacteria were treated in PGNO liquid for 1 h. (**b**) CFU number of *S. aureus* treated with differently diluted PGNO DI water and 0.5 mM phosphate buffer. Bacteria were treated in PGNO liquid for 1 h. (**c**) Number of germinated *F. oxysporum* spores treated with differently diluted PGNO DI water and 0.5 mM phosphate buffer for 1 h. Each value represents the average ± standard deviation of 3–9 replicate measurements. These replicate measurements were used for statistical analysis. *p < 0.05, **p < 0.01.

In order to analyze the potential of PGNO water and phosphate buffer for inactivating microorganisms, we incubated bacteria and fungal spores in PGNO-treated water and phosphate buffer. There was a 5–8 log reduction in CFU number for *E. coli* incubated in un- and half- diluted PGNO-treated water and 0.5 mM phosphate buffer for 1 h (Fig. 8a). PGNO-treated water and phosphate buffer diluted 10 times inactivated about 50% of *E. coli* cells (0.2–0.3 log reduction in CFU number) within 1 h (Fig. 8a). In the treatment of *S. aureus*, 7–8 log reduction in CFU number was observed in the treatment with un- and half- diluted PGNO-treated water, and about 0.6 log reduction (about 50% of bacterial cells) in the treatment with 10 times diluted PGNO water (Fig. 8b). The CFU number of *S. aureus* was reduced up to about 2 log scale in the treatment with un- and half- diluted PGNO-treated 0.5 mM phosphate buffer for 1 h, and up to 0.1–0.4 log scale (30–60%) in the treatment with 10 and 30 times diluted PGNO phosphate buffer, compared to control (Fig. 8b).

The number of germinated *F. oxysporum* spores was significantly reduced (60–80%) when spores were incubated in un-diluted PGNO-treated water and phosphate buffer for 1 h (Fig. 8c). Spores treated in half diluted PGNO-treated 0.5 mM phosphate buffer were germinated at significantly lower rate (20% reduction) than control (Fig. 8c, Supplementary Table S1). However, there was no dramatic difference in the percentage of germination of spores treated with 10 and 30 times diluted PGNO water and phosphate buffer (Fig. 8c).

## Discussion

Our results suggest several implications for the dynamics of NO level in PGNO treated liquid. First, the removal of O_2_ in liquid is critical for enhancing NO assimilation into liquid. Our results show that NO concentration in liquid was more dramatically increased when N_2_ gas was injected longer (for O_2_ removal), regardless of the kind of liquid (water or phosphate buffer). Although the removal of O_2_ is efficient in increasing NO assimilation, O_2_ removal by N_2_ injection may not be practical in the agricultural application because of economic expenses and the effect of injected N_2_. PGNO treated liquid without O_2_ removal was applied to plants in our experiments. Since the working NO concentration enhancing plant vitality is important and can be obtained without O_2_ removal, it may not be essential in a large scale or practical application. Interestingly, NO was more slowly assimilated into higher than lower concentration of phosphate buffer (higher buffering strength). This might be because potassium phosphate can react with NO. In addition, slightly higher deposition of NO in water than phosphate buffer could be also explained by the reaction of NO with potassium phosphate. Secondly, our results showed that NO decay in PGNO-treated water and buffer (after 50 min PGNO injection) followed exponential change, and NO was not completely perished in 1 L liquid after 16 h without further injection. Decrease in NO level was slightly faster during the early stages in both water and phosphate buffer. This may be a result of the reaction of NO with other species in PGNO liquids. Slightly faster decrease in NO level in PGNO than pure NO gas-treated water during the early stages also indicates that species other than NO may be present, and react with NO in PGNO-treated liquids. As shown in our reults, H_2_O_2_, NO_2_^−^, and NO_3_^−^ as well as NO were detected in PGNO treated water and buffer. The O_2_ purging (by N_2_ injection) before PGNO injection did not affect the rate of decrease in NO level in PGNO-treated water and buffer, because NO kept reacting with oxygen continuously assimilated from the air into the liquid placed in an open container during microwave plasma generation.

Ions and reactive species, such as H_2_O_2_, NO_2_^−^, and NO_3_^−^, were also detected in PGNO-treated water and phosphate buffer in our study. The most dominant radicals in the microwave nitrogen plasma are excited nitrogen molecules, which exist in abundance in the metastable state of N_2_(A_3_Σ_u_^+^)^31^. Since our NO generation system may not be perfectly sealed, humid air could continuously be assimilated into the system, providing water molecules. Hydroxyl molecules (OH) are generated from the dissociation of water molecules when they are in contact with excited nitrogen molecules, according to (1). Meanwhile, hydroxyl molecules combine together forming hydrogen peroxide molecules (2). The nitrogen monoxide molecules (NO) come into contact with the hydroxyl molecules, forming nitrous acid, HNO_2_ (3). On the other hand, the nitrous acid HNO_2_ from NO and OH combination is quenched, due to the reaction (4). The nitrogen dioxide (NO_2_) thus formed may be eliminated through the reaction (5).

1 $${{\rm{N}}}_{2}({{\rm{A}}}_{3}{\sum }_{{\rm{u}}}^{+})+{{\rm{H}}}_{2}{\rm{O}}\to {\rm{OH}}+{{\rm{N}}}_{2}+{\rm{H}}$$

2 $${\rm{OH}}+{\rm{OH}}+{\rm{M}}\to {{\rm{H}}}_{2}{{\rm{O}}}_{2}+{\rm{M}},\,{\rm{where}}\,{\rm{M}}\,{\rm{is}}\,{\rm{a}}\,{\rm{neutral}}\,{\rm{particle}}$$

3 $${\rm{NO}}+{\rm{OH}}\to {{\rm{HNO}}}_{2}$$

4 $${{\rm{HNO}}}_{2}+{\rm{OH}}\to {{\rm{H}}}_{2}{\rm{O}}+{{\rm{NO}}}_{2}$$

5 $${{\rm{NO}}}_{2}+{\rm{OH}}\to {{\rm{HNO}}}_{3}$$

The molecules of H_2_O_2_, HNO_2_, and HNO_3_ may dissolve into liquid, to generate H_2_O_2_, NO_2_^−^, and NO_3_^−^. It is highly possible that these components have contributed to generate fertilizing and sterilizing effects, because NO_3_^−^ is a well known nitrogen nutrient to plants, and H_2_O_2_ and NO_2_^−^ are harmful to microbes. In PGNO-treated water and phosphate buffer, NO and H_2_O_2_ as reactive species are involved in both promoting plant vitality and inactivating microbes, whereas NO_2_^−^ may generate antimicrobial effect, and NO_3_^−^ has acted as nutrient to plants.

The fertilization property and sanitation of culture media are very important in plant culture systems. Since plants and microorganisms are very different in cell structure and properties, the same amount of reactive species generated by plasma can possibly produce different or contrary outcomes on plant and microorganism. Different plasma effects on eukaryote and prokaryotic cells have occasionally been demonstrated in studies^32,33^. Dual effects (enhancement of plant vitality and inactivation of microbes) of PGNO-treated water and phosphate buffer were not simultaneously observed in our study. From our results, 10–30 times diluted PGNO-treated water (about 2.8–8.5 µM NO) and 0.5 mM phosphate buffer (about 2.5–7.5 µM NO) seem to enhance both seedling growth and tolerance to drought stress. Undiluted PGNO water and buffer inhibited the growth of plants because NO, H_2_O_2_, NO_2_^−^, and NO_3_^−^ detected in PGNO liquids are toxic to plant cells in large amount, and the liquids are too acidic (lower than pH 3.0). Acidity of 10–30 times diluted PGNO water and buffer (pH 3.5–5.5) seems to show no adverse effect on plant growth. In addition, NO and NO_3_^−^ may possibly have contributed relatively more to the enhancement of plant vitality than H_2_O_2_ and NO_2_^−^ because NO_3_^−^ is a major nitrogen nutrient and NO can act as a activating signal. Acidity and the level of NO, H_2_O_2_, NO_2_^−^, and NO_3_^−^ in 10–30 times diluted PGNO liquids may not be enough to kill microbes completely as demonstrated in our study. The highest antimicrobial activity was observed in undiluted PGNO liquids, probably due to the high level of NO, H_2_O_2_, and NO_2_^−^, and acidic pH. Treatment time (1 h) may be not sufficient for complete antimicrobial activity. Longer incubation or treatment may increase the level of microbial inactivation because the effect of incubation time in plasma-treated media on microbial inactivation is occasionally observed in studies^34^. However, long treatment is not likely to be practical in many applications. Microbial inactivation and fertilization by PGNO water and buffer can be separately achieved if we consider to apply those to small size of stock (concentrated) nutrient solutions. For example, microbes in a small stock solution are inactivated by direct PGNO treatment, and then the PGNO treated stock solution is diluted for applying to plants.

A promising effect of PGNO observed in our study is that 10–30 times diluted PGNO water significantly increase spinach tolerance to water deficient stress, and induce the expression of PR gene (encoding a defense protein against pathogen attack). Slight increase in the tolerance to drought stress was also observed in plants treated with 10–30 times diluted PGNO buffer although the difference was not significant. These results may provide useful information on stress regulation, particularly in these days in which global warning becomes a major issue worldwide. The elevated tolerance and PR10 gene expression may be a consequence of the enhanced plant growth and vitality by PGNO solutions. On the other hand, PGNO itself can trigger defense signaling in plant, since NO is a well-known signaling molecule in the regulation of drought and pathogen stresses^22–24^. Expression of many stress related genes can be induced by intracellular and exogenous NO^22^. NO causes *S*-nitrosylation of proteins such as transcription factors and other signaling pathway proteins, and *S*-nitrosylated (functionally activated) proteins may trigger or repress the expression of stress related genes such as PR, leading to the promotion of plant tolerance^35–37^. Intruiguingly, our results show that PGNO treated water seems to induce tolerance to drought stress and PR10 expression more greatly than PGNO treated buffer. Level of NO is not likely to be a reason because no significant difference is observed between PGNO treated water and buffer. Since potassium phosphate is known to play a role in stress regulation in plants^38,39^, additional potassium phosphate may possibly reduce the effect of PGNO as a stress signal. Further study on this will be needed.

In conclusion, our results suggest that PGNO treatment may be able to help sanitation of water and buffer and enhance the fertilizing efficiency of water and buffer, by which plants can be more tolerant to stresses. PGNO (microwave plasma-generated gas) can be efficiently used in agricultural water management, applicable to hydroponic culture system and plant factory. For example, microbial contamination in water or even plant nutrient stock solution is removed by PGNO treatment, and then the sanitated water or nutrient stock solution is diluted and used for plant fertilization. However, action mechanisms of PGNO are still not clear. Our results suggest that NO and NO_3_^−^ may have contributed to the enhancement of plant growth and stress tolerance, and H_2_O_2_ and NO_2_^−^ may play more role in antimicrobial effect.

## Methods

### Materials for sensors

Chloroplatinic acid hexahydrate, 3-aminobenzoic acid (ABA), lead(II) acetate trihydrate, iron(II) sulfate heptahydrate, potassium dioxide, dimethyl sulfoxide (DMSO), silver wire (250 μm in diameter), and platinum wire (76 μm in diameter) were purchased from Sigma (St. Louis, MO, USA). Tungsten wire (100 μm in diameter) was purchased from Alfa Aesar (Ward Hill, MA, USA). Calcium chloride and silver conducting epoxy paste were products of Junsei (Tokyo, Japan) and ASAHI (Tokyo, Japan), respectively. Methyltrimethoxysilane (MTMOS) and (Heptadecafluoro-1,1,2,2-tetrahydrodecyl) trimethoxysilane (17FTMS) were purchased from Fluka (Buchs, Switzerland) and Gelest (Tullytown, PA), respectively. Nitric oxide (99.99% and 87,500 ppm) and nitrogen (99.99%) gases were purchased from Dong-A Scientific (Seoul, Korea). Other solvents and chemicals were analytical-reagent grade. Distilled water of 18.2 MΩ cm resistivity was used for preparing all aqueous solutions.

### Microwave plasma torch system and treatment of water and buffer with plasma generated gas

Figure 1a and b show the microwave plasma system generating nitric oxide, and treatment of liquids with gas generated from plasma system. The system configuration has been well described in previous study^40^. Microwave (2.45 GHz) radiated from a magnetron passes through a circulator, power monitor, and three-stub tuner, and then is guided through a tapered waveguide, entering a discharge tube made of quartz. In order to generate a microwave plasma torch, nitrogen (10 lpm) and oxygen (400 sccm) gases controlled by mass flow meter were mixed and injected into the system, and a microwave power of 400 W was applied. In the discharge tube, a plasma torch (temperature; 6,000 K; plasma density, 10^13^/cm^3^) was generated. Gas generated from the torch flame was cooled down by passing through metal pipe wrapped with water tubing (Fig. 1a), and then injected into 1 L of deionized (DI) water or 0.5 and 50 mM potassium phosphate buffer (pH 6.0), as shown in Fig. 1a and b. Phosphate buffer of pH 5.0–6.0 is one of common buffers used in many plant nutrient and tissue culture solutions. We used potassium phosphate buffer rather than plant nutrient solution because nutrient solution contained many components, and the chemistry of solution would be more complicated after PGNO treatment. Therefore, we chose to start with a simple background buffer of plant nutrient solution, potassium phosphate buffer (pH 6.0) to make the system simpler.

### Preparation of amperometric NO and H_2_O_2_ microsensors

To prepare a cone-shaped platinum (Pt) microelectrode^41^, Pt wire (76 μm in diameter) was first immersed in an etching solution containing 1.2 M calcium chloride in water/acetone (2:1, v/v) and then electrochemically etched for 60 s by applying a 5 V amplitude AC voltage at a frequency of 60 Hz between the Pt wire electrode and a Pt counter electrode using a CH Instruments 760D bipotentiostat (Austin, TX,USA). The etched Pt wire was then inserted into a pulled borosilicate glass capillary (600/550 μm o.d./i.d.; A-M Systems; Sequim, WA, USA). The glass capillary was shielded around the wire using a flame torch, and rinsed with distilled water. To increase the effective surface area of the electrode, the Pt electrode was platinized to form mesoporous platinum black (Pt-B) on Pt surface in 3% chloroplatinic acid hexahydrate and 0.03% lead(II) acetate trihydrate (wt/wt in water) by cycling the potential from +0.6 to −0.35 V (vs. Ag/AgCl) at a scan rate of 20 mV s^−1^ using a Compactstat (Ivium Technology, Netherland). The Ag/AgCl wire (250 μm in diameter) as a reference electrode was formed by immersing the Ag wire in an aqueous 0.3 M FeCl_3_ solution for 15 min and was then coiled outside the glass capillary.

To fabricate nitric oxide (NO) microsensors^42^, the abovementioned Pt-B/Pt electrode was modified with perfluorinated xerogel-derived gas-permeable membrane by dip-coating the sensor tip into a sol solution for 6 times at 10 minutes interval. A silane sol solution was prepared by dissolving 300 μL of MTMOS and 75 μL of 17FTMS in 300 μL of ethanol. The solution was mixed with 80 μL of water followed by 5 μL of 0.5 M HCl for 1.5 h. The perfluorinated xerogel-modified electrode was then allowed to cure for 12 h under ambient conditions.

The hydrogen peroxide (H_2_O_2_) microsensors were prepared by modifying the Pt-B/Pt electrode with poly(3-aminobenzoic acid) (PABA) thin film as an H_2_O_2_ permselective membrane^43^. The PABA membrane was readily formed by electropolymerization in 0.1 M 3-aminobenzoic acid (ABA) and 0.5 M sulfuric acid solution by cycling the potential from −0.1 to +0.9 V at a scan rate of 0.1 mV s^−1^.

### *In situ*, real-time monitoring of NO and H_2_O_2_ during plasma treatment time

To evaluate the analytical performance of the NO and H_2_O_2_ microsensors, amperometric signals were collected using a Compactstat in phosphate-buffered saline (PBS; 0.05 M, pH 6.0). The standard solutions of NO and H_2_O_2_ were freshly prepared for every measurement. The potentials applied to the sensors were different; while the NO sensor measured NO oxidation currents at +0.8 V (vs. Ag/AgCl), the H_2_O_2_ sensor measured oxidation currents of H_2_O_2_ at +0.3 V (vs. Ag/AgCl).

### Measurement of gaseous nitric oxide level, dissolved oxygen (DO) level, and pH

The concentration of nitric oxide in gas generated by microwave plasma torch was measured using nitric oxide gas measuring instrument (UniGas 1000+, EUROTRON, Italy). The level of dissolved oxygen (DO) in liquids was measured using dissolved oxygen meter (PDO-519, Lutron electronic, Taiwan). The pH of liquids was measured using a portable pH meter (Eutech Instruments, Singapore). All measurements were repeated at least three times.

### Ion analysis

Several positive and negative ions in liquids treated with plasma-generated gas were quantitatively analyzed using ion chromatography. After plasma generated gas was injected into 1 L of DI water and 0.5 mM potassium phosphate buffer without O_2_ purging for 50 min, treated water and phosphate buffer were filtered (0.5 μm pore size). Filtered liquid was then analyzed using chromatograph ICS-3000 (Thermo Scientific Dionex, Sunnyvale, CA, USA). Methanesulfonic acid (MSA, 20 mM) and potassium hydroxide (KOH, 12 mM, 30 mM) were used as effluent for cation and anion analysis, respectively. Filtered sample (25 µL) was injected into Ionpac AS20 (4 × 250 mm) column (Thermo Scientific Dionex, Sunnyvale, CA, USA) kept at 30 °C. Flow rate of effluent was 1 mL/min, and sequencial running of KOH was performed for anion analysis; 0–8 min 12 mM KOH, 8–12 min 30 mM KOH, 12–17 min 30 mM KOH, 17–18 min 12 mM KOH, and 18–20 min 12 mM KOH. The level of ions in liquids was detected using suppressed conductivity detection system (4 mm format, recycle mode).

### Assay for seed germination and growth

Plasma-generated gas (PGNO) was injected into DI water (1 L) and 0.5 mM potassium phosphate buffer (pH 6.0, 1 L) without O_2_ removal (N_2_ injection) for 50 min. DI water and phosphate buffer treated with plasma-generated gas were then immediately diluted 2, 10, and 30 times. In order to reduce the time consumed for the dilution process, treated water and buffer were immediately added into new DI water or 0.5 mM phosphate buffer already placed in a 100 ml scaled beaker. Non-treated DI water and 0.5 mM phosphate buffer were used as control. Non-treated (control) and treated (1x, 1/2x, 1/10x, 1/30x diluted) water and buffer were applied to seeds and plants.

For germination assay, spinach seeds (50 seeds per treatment) were soaked in 10 ml of non-treated DI water (control), non-treated 0.5 mM phosphate buffer (control), treated (1x, 1/2x, 1/10x, 1/30x diluted) DI water, or treated (1x, 1/2x, 1/10x, 1/30x diluted) phosphate buffer for 30 min. After soaking, seeds were placed on 2 layers of wet filter paper in a petri-dish, and the petri-dish was incubated in a plant growth chamber (25 °C, 50% humidity, 16 h light and 8 h dark). The number of germinated seeds was counted every day for a week. Three replicate measurements per treatment were performed.

To analyze the effects on plant growth, 50 seeds (per treatment) were planted in each pot (100 mm diameter × 50 mm height) containing vermiculite, and the pots were incubated in a plant growth chamber (25 °C, 50% humidity, 16 h light and 8 h dark). Every once a week, 100 ml of non-treated DI water (control), non-treated 0.5 mM phosphate buffer (control), treated (1x, 1/2x, 1/10x, 1/30x diluted) DI water, or treated (1x, 1/2x, 1/10x, 1/30x diluted) phosphate buffer was applied to each pot. Additional DI water (100 ml) was applied to each pot once a week to prevent drying. Plants were harvested after 5 weeks, and the number of survived plants was counted. Length of shoot and root was also measured in individual plant. Then, plants were dried at 60 °C for 3–4 days, and total dry weight of plants collected from each pot was measured. The average dry weight of individual plant was calculated by dividing total dry weight by number of plants. Each treatment was performed in 3 replicate pots.

### Assay for tolerance to drought stress

Spinach seeds were planted in pots (100 mm diameter x 50 mm height) containing vermiculite (50 seeds per pot), and the pots were incubated in a plant growth chamber (25 °C, 50% humidity, 16 h light and 8 h dark). Non-treated DI water (control), non-treated 0.5 mM phosphate buffer (control), treated (1x, 1/2x, 1/10x, 1/30x diluted) DI water, or treated (1x, 1/2x, 1/10x, 1/30x diluted) phosphate buffer (100 ml each) were applied to each pot once a week for 4 weeks. Pots were additionally watered (100 ml per pot) once a week to prevent drying. Then, seedlings were then kept under no water condition for 2 weeks. Plants were harvested after total 6 weeks, and dried at 60 °C for 3–4 days. The total dry weight of plants collected from each pot was measured, and the average dry weight of individual plant was calculated by dividing total dry weight by number of plants. Each treatment was performed in 4 replicate pots.

### QPCR for quantifying the transcription level of PR10 gene

The level of PR10 mRNA was measured in spinach plants grown for 4 weeks under treatment with water or phosphate buffer pre-treated with microwave plasma-generated gas. Plants grown for 4 weeks were ground into powder in liquid nitrogen, and total RNA was extracted using the TaKaRa RNAiso Plus kit (TaKaRa Bio, Tokyo, Japan), as described in the previous study^44^. The RNA concentration was measured using a nanodrop (Biotek, Winooski, VT, USA), and then 1 µg RNA was treated with RNase-free DNase (Promega, Madison, WI, USA) at 37 °C for 1 h, to remove genomic DNA. The same amount of RNA (120 ng) was used to synthesize cDNA using the miScriptII PCR System, following the manufacturer’s protocol (Qiagen, Valencia, CA, USA). PCR conditions were as follows: 60 min at 37 °C, and then 5 min at 95 °C. PR10 gene was amplified and quantified at every thermal cycle using iQ SYBR Green Supermix (Bio-Rad, Hercules, CA, USA) and the CFX96^TM^ real time RT-PCR system (Bio-Rad, Hercules, CA, USA). The sequence of primers for amplifying PR10 cDNA was as follows: TGGCGGTCCATACAAGTACA (forward) and AAACGACATCGCCCTTAGTG (reverse). PR10 mRNA level was normalized by the quantity of Actin (a reference gene) mRNA. The primer sequences for amplifying Actin cDNA were GAGGCACCATTGAACCCTAA (forward) and AGGGCGTAACCCTCGTAGAT (reverse). The relative expression level of PR10 mRNA in plants treated with water or buffer pre-treated with plasma-generated gas was expressed as relative ratio compared to that of control.

### Test for antimicrobial activity of liquids treated with plasma-generated gas

The antimicrobial activity of water and buffer treated with plasma-generated gas was assessed using 2 bacterial (*Escherichia coli*, *Staphylococcus aureus*) and 1 fungal (*Fusarium oxysporum f.sp. lycopersici*) species. Two or three bacterial colonies were suspended in 1.8 ml LB liquid, and then 10 μl of the suspension was inoculated into 15 ml LB liquid. After inoculation, culture tubes were incubated for 16 and 20 h for *E. coli* and *S. aureus*, respectively, with shaking. Then, bacterial cells were pelleted down by centrifugation (5 min at 3,134 × *g*), washed with sterile saline once, and resuspended in new saline. Optical density (OD) of bacterial suspension was adjusted to 0.1 and 0.4 for *E. coli* and *S. aureus*, respectively, in order to get 10^8^ bacterial cells per ml concentration. *F. oxysporum* spores were prepared as follows: 200 ml Vogel’s minimal media inoculated with pieces of fungal hypha was incubated at 28 °C for 3–4 days with shaking, and fungal culture was filtered through 3 layers of miracloth to get spores. After washing with deionized water once, spores were resuspended in DI water, to make the concentration 10^8^ spores per ml. After the concentration was adjusted, 1 ml of bacterial cell or fungal spore suspension was placed in a microfuge tube, and centrifuged at 3,134 × *g* for 5 min. After liquid was discarded, bacterial or fungal cell pellets were resuspended in 1 ml of water, or buffer treated with plasma-generated gas. Tubes were incubated for the indicated time, and then 100 μl of serially diluted suspension was plated on LB agar (for bacteria) and Potato Dextrose Agar (PDA, for fungus) plates. Plates were incubated at 37 and 25 °C for bacteria and fungus, respectively, and CFU and germinated spore number were counted after 1–2 days.

### Statistical analysis

All data were indicated as average and standard deviation of 3 or more replicate measurements. Student’s *t* test was performed to determine the significance between data points. Significant differences were established at *p* < 0.05 or *p* < 0.01 (*denotes *p* < 0.05, and ** denotes *p* < 0.01).

## Supplementary information

Supplementary information

Supplementary table S1
