# Supplementary material for: Dynamics of nitric oxide level in liquids treated with microwave plasma-generated gas and their effects on spinach development
Source: Sci Rep. 2019 Jan 30;9:1011. doi: 10.1038/s41598-018-37711-3 (PMC6353906; doi:10.1038/s41598-018-37711-3)
Supplement: Supplementary file 1 — Supplementary information [file 41598_2018_37711_MOESM1_ESM.pdf]

Supplementary Information

Dynamics of nitric oxide level in liquids treated with microwave plasma-generated gas and their effects on spinach development

Min-Ho Kang[1,2†], Seong-Sil Jeon[1,2†], So Min Shin[3], Mayura Veerana[1], Sang-Hye Ji[1††], Han-Sup Uhm[1,4], Eun-Ha Choi[1,2], Jae Ho Shin[3*], and Gyungsoon Park[1,2*]

[1] Plasma Bioscience Research Center, Kwangwoon University, Seoul, 01897, Korea

[2] Department of Electrical and Biological Physics, Kwangwoon University, Seoul, 01897, Korea

[3] Department of Chemistry, Kwangwoon University, Seoul, 01897, Korea

[4] New Industry Convergence Technology R&D Center, Ajou University, Suwon 16499, Korea

*Corresponding authors:

Gyungsoon Park

Phone: +82-2-940-8324

Fax: +82-2-940-5664

Email: gyungp@kw.ac.kr

Jae Ho Shin

Phone: +82-2-940-5627

Email: jhshin@kw.ac.kr

† Equally contributed to this work

†† Present address:

Plasma Technology Research Center, National Fusion Research Institute, Gunsan-si, Jeollabuk-Do, 54004, Republic of Korea

1. Electrooxidation process of nitric oxide on a platinum working electrode.

The electrochemical oxidation of NO on a polycrystalline platinum electrode proceeds via the following reactions.[S1-S3] In the first step (Eq. S1) an electron is transferred from NO to the electrode, generating an oxidation current. Since nitrosonium ion (NO^+) is a relatively strong Lewis acid, it is converted to nitrite (NO_2^-) in the presence of hydroxide (OH^-) (Eq. S2).

$$NO \rightarrow NO^+ + e^- \quad (S1)$$

$$NO^+ + OH^- \rightarrow HNO_2 \quad (S2)$$

Ultimately NO_2^- is further oxidized to nitrate (NO_3^-), the final product of NO's electrochemical oxidation, resulting in the additional transfer of two electrons to the platinum electrode:

$$HNO_2 + H_2O \rightarrow NO_3^- + 3H^+ + 2e^- \quad (S3)$$

2. Calculation of the concentration for a standard NO solution (adapted from ref. S4).

The molarity of NO (C_{NO}, mol·L^{-1}) in phosphate-buffered saline (PBS; containing 3.9 mM NaH_2PO_4, 6.1 mM Na_2HPO_4, 2.7 mM KCl, and 138 mM NaCl, pH 6.0) can be calculated from Henry's law:

$$C_{NO} = P_{NO} \cdot S_{NO} \tag{S4}$$

where P_{NO} is the pressure of NO (mmHg), and S_{NO} is the solubility of NO in water (cm^3). Alternatively, C_{NO} and S_{NO} in water may be calculated as C_{NO} and S_{NO} in saline or PBS. The pressure of NO (P_{NO}) may be expressed as follows.

$$P_{NO} = (P_{tot} - P_w)(G_{NO})10^{-6} \tag{S5}$$

where P_{tot} and P_w are the total pressure (760 mmHg) of the gas mixture and the partial pressure of water vapor (23.8 mmHg) [S5] at 25°C, respectively. In addition, G_{NO} is the NO concentration obtained from a gas cylinder (ppm). For our studies, S_{NO} at 25°C is 0.0432 cm^3.[S6] Finally, converting the units of pressure from mmHg to mol·L^{-1} yields the following expression:

$$C_{NO} = (760 - 23.8)\frac{133.322 \times 10^{-3}}{8.314(273.15 + 25)}(G_{NO})10^{-6}(0.0432) \tag{S6}$$

Therefore, when PBS is purged with 87,500 ppm of NO gas (G_{NO}), C_{NO} is about 150 µM.

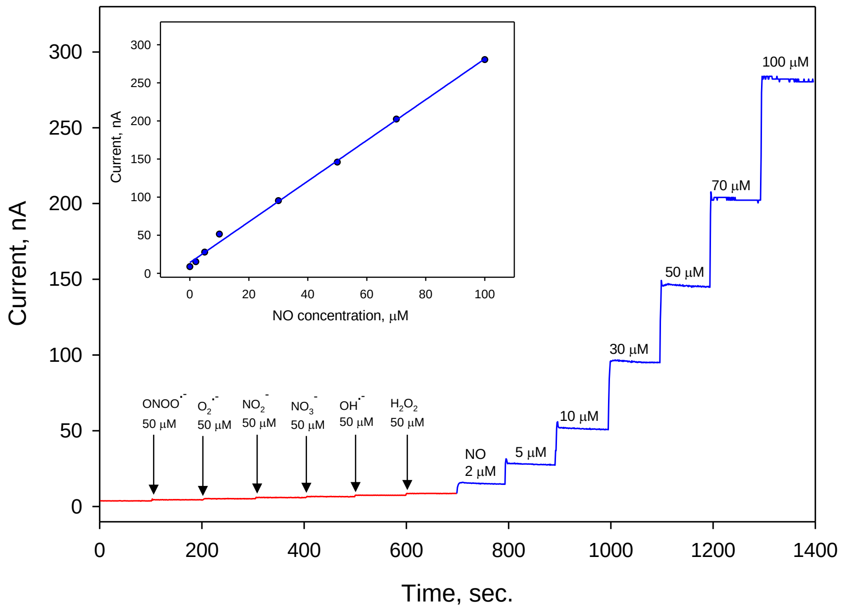

Supplementary Figure S1. Dynamic response of a nitric oxide sensor using a Pt-B/Pt microelectrode modified with a perfluorinated xerogel membrane towards a series of interfering species (red) and nitric oxide (blue). Currents were recorded at an applied potential of +0.8 vs. Ag/AgCl in PBS (0.01 M, pH 6.0). Inset represents the calibration curve.

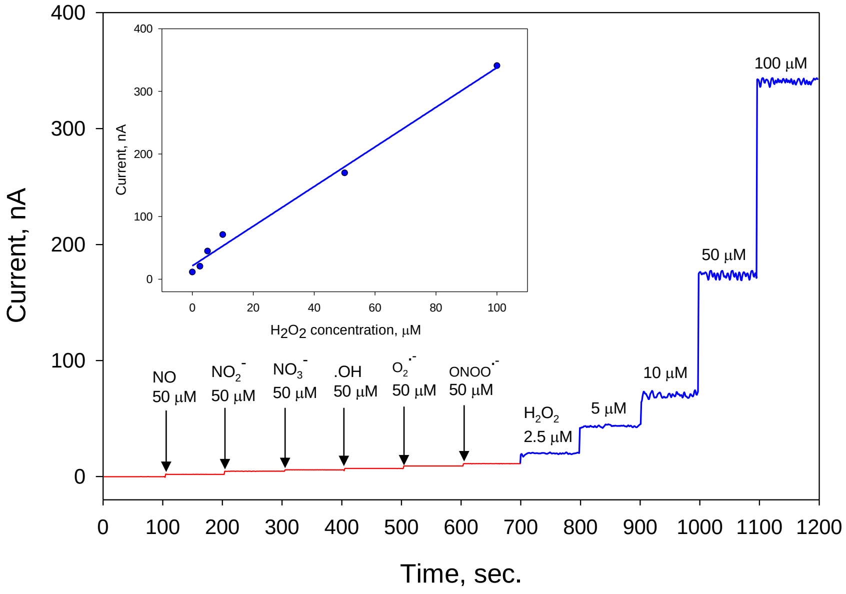

Supplementary Figure S2. Dynamic response of a hydrogen peroxide sensor using a platinum microelectrode electropolymerized with poly (3-aminobenzoic acid) (PABA) permselective membrane towards a series of interfering species (red) and hydrogen peroxide (blue). Currents were recorded at an applied potential of +0.3 vs. Ag/AgCl in PBS (0.01 M, pH 6.0). Inset represents the calibration curve.

SI REFERENCES

S1. Lee, Y.; Oh, B. K.; Meyerhoff, M. E. *Anal. Chem.* **2004**, *76*, 536-544.

S2. Bedioui, F.; Trevin, S.; Devynck, J. *Electroanalysis* **1996**, *8*, 1085-1091.

S3. de Vooys, A. C. A.; Beltramo, G. L.; van Riet, B.; van Veen, J. A. R.; Koper, M. T. M. *Electrochim. Acta* **2004**, *49*, 1307-1314.

S4. Kitamura, Y.; Uzawa, T.; Oka, K.; Komai, Y.; Ogawa, H.; Takizawa, N.; Kobayashi, H.; Tanishita, K. *Anal. Chem.* **2000**, *72*, 2957-2962.

S5. Dean, J. A. *Lange's Handbook of Chemistry*, 15th ed.; McGraw-Hill: New York, 1999; pp 5.28.

S6. Dean, J. A. *Lange's Handbook of Chemistry*, 15th ed.; McGraw-Hill: New York, 1999; pp 5.6-5.7.
